# Cognitive and neural abnormalities: working memory deficits in bipolar disorder offspring

**DOI:** 10.1017/S0033291725001060

**Published:** 2025-05-02

**Authors:** Ye Xie, Wenjin Zou, Yuanqi Shang, Weicong Lu, Xiaoyue Li, Qi Chen, Robin Shao, Yixuan Ku, Kangguang Lin

**Affiliations:** 1 School of Psychology, Shenzhen University, Shenzhen, P.R. China; 2 Philosophy and Social Science Laboratory of Reading and Development in Children and Adolescents (South China Normal University), Ministry of Education, Guangzhou, P.R. China; 3 Guangdong Provincial Key Laboratory of Brain Function and Disease, Center for Brain and Mental Wellbeing, Department of Psychology, Sun Yat-sen University, Guangzhou, P.R. China; 4 Department of Affective Disorder, The Affiliated Brain Hospital, Guangzhou Medical University, Guangzhou, P.R. China; 5 State Key Laboratory of Brain and Cognitive Sciences, Department of Psychology, University of Hong Kong, Hong Kong, P.R. China; 6 Peng Cheng Laboratory, Shenzhen, P.R. China; 7 Key Laboratory of Neurogenetics and Channelopathies of Guangdong Province and the Ministry of Education of China, Guangzhou Medical University, Guangzhou, P.R. China; 8 Department of Neurology, Lecong Hospital of Shunde, Foshan, Guangdong, China

**Keywords:** BD offspring, bipolar disorder, functional magnetic resonance imaging, prefrontal cortex, working memory

## Abstract

**Background:**

Offspring of parents with bipolar disorder (BD offspring) face elevated risks for emotional dysregulation and cognitive deficits, particularly in working memory. This study investigates working memory deficits and their neural correlates in BD offspring.

**Methods:**

We assessed 41 BD offspring and 25 age-matched healthy controls (HCs) using a spatial N-back task and task-related functional magnetic resonance imaging (fMRI).

**Results:**

Compared to HCs, BD offspring exhibit reduced accuracy and lower signal-detection sensitivity (*d*′) on the 1-back task. fMRI reveals hyperactivation in the right intracalcarine cortex/lingual gyrus (ICC/LG) in BD offspring, particularly during the 1-back condition. Psychophysiological interaction (PPI) analyses show reduced connectivity between the right ICC/LG and the left postcentral gyrus in BD offspring as task load increases from 0-back to 1-back. This connectivity positively correlates with 1-back task performance in HCs but not in BD offspring. Additionally, using bilateral dorsolateral prefrontal cortex (DLPFC) as regions of interest, PPI analyses show diminished condition-dependent connectivity between the left DLPFC and the left superior frontal gyrus/paracingulate cortex, and between the right DLPFC and the left postcentral gyrus/precentral gyrus in BD offspring as the task load increases.

**Conclusions:**

These findings suggest that BD offspring exhibit working memory deficits and impaired neural connectivity involving both sensory processing and higher-order cognitive systems. Such deficits may emerge at a genetically predisposed stage of bipolar disorder, underscoring the significance of early identification and intervention strategies.

## Introduction

Bipolar disorder (BD) is a complex mental illness distinguished by alternating episodes of mood disturbance, encompassing depressive, manic, and hypomanic states (Grande, Berk, Birmaher, & Vieta, 2016; McIntyre et al., 2020). Its etiology is underpinned by a substantial genetic component, with heritability estimates highlighting the influence of genetic factors in its development (Jones & Bentall, 2008; McGuffin et al., 2003; Morey et al., 2023). Individuals who are offspring of BD patients, henceforth referred to as BD offspring, are identified to be at an elevated genetic risk for the disorder (Lichtenstein et al., 2009). Longitudinal research, including our own, has indicated that approximately 20% of BD offspring eventually develop BD, a rate significantly higher than the general population’s incidence rate, which stands at 5–10 times lower (Au et al., 2021; Duffy, Goodday, Keown-Stoneman, & Grof, 2019; Li et al., 2021; Liu et al., 2024; Mesman et al., 2013; Zou et al., 2022). It is hypothesized that genes associated with BD may initially impact cognitive functions, brain structure, and neural processes, potentially serving as intermediate phenotypes that predispose at-risk individuals to the disorder (Hou et al., 2022; Lu et al., 2024; de Sá et al., 2016; Wu et al., 2025).

Within the spectrum of cognitive domains affected in BD, working memory (WM) deficits are a consistent finding, observed across various mood states, including periods of clinical remission, and are also present in unaffected first-degree relatives of individuals with BD (Saldarini, Gottlieb, & Stokes, 2022; Soraggi-Frez et al., 2017; Valencia-Echeverry et al., 2022). This body of evidence supports the notion that WM deficits may constitute endophenotypes for BD. The neural substrates of WM deficits in BD have been the subject of extensive investigation. Research has uncovered abnormalities across a spectrum of brain regions, from frontoparietal networks to primary sensory areas, with activation patterns varying in accordance with task demands and the mood states of individuals with BD (Adler et al., 2004; Dell’Osso et al., 2015; Saldarini et al., 2022; Thermenos et al., 2010). Despite these findings, the precise mechanisms underpinning WM deficits in BD offspring remain to be fully understood. BD offspring presenting with subthreshold symptoms are of particular interest, as they may represent a vulnerable group at risk for the future onset of BD, thereby offering a unique opportunity to identify potential biomarkers for the disorder’s development.

The present neuroimaging study, employing a spatial N-back task, aimed to delineate the neural correlates of WM deficits in BD offspring relative to healthy controls (HCs). We posited that BD offspring would exhibit WM impairments in comparison to HCs and that these impairments would be linked to aberrant activation patterns as well as atypical functional connectivity within networks implicated in visual processing, sensory integration, and higher-order cognitive functions.

## Method

### Participants

This study is a part of the Recognition and Early Intervention on Prodromal Bipolar Disorder (REI-PBD) Project and has received ethical approval from the Institutional Research Board of the Affiliated Brain Hospital of Guangzhou Medical University. Written informed consent was obtained from all participants and their guardians (if under the age of 18). The study was conducted following the Declaration of Helsinki and approved by the institutional ethics committee of the Affiliated Brain Hospital of Guangzhou Medical University. The methodology of our project has been detailed in previous publications (Lin et al., 2015, 2018; Lu et al., 2024; Zou et al., 2022), but the data in this study have not been published or released before.

Participants underwent comprehensive symptom assessments utilizing the Hamilton Depression Rating Scale (HAMD), the Young Mania Rating Scale (YMRS), the Hamilton Anxiety Rating Scale (HAMA), and the Brief Psychiatric Rating Scale (BPRS) to evaluate the severity of depressive, manic, anxious, and psychotic symptoms, respectively.

Eligible offspring (BD offspring) were those with at least one biological parent diagnosed with bipolar disorder (BD I or II). Recruitment was facilitated through advertisements, referrals from psychiatrists, and word-of-mouth. The family history of psychiatric disorders was validated using the Family Interview for Genetic Studies (FIGS). Participants aged under 18 years were assessed using the Schedule for Affective Disorders and Schizophrenia for School-Aged Children: Present and Lifetime Version (K-SADS-PL DSM-IV), while those 18 years and older were evaluated using the Structured Clinical Interview for DSM-IV Axis I Disorders, Patient Edition (SCID-I/P), to rule out any current or past psychiatric disorders.

For both BD offspring and the comparison group of healthy controls (HCs), exclusion criteria included physical conditions precluding neuropsychological testing, substance abuse within the past 3 months, the use of psychoactive medications or thyroxine, and the presence of severe medical conditions.

### fMRI task

**Figure 1.**
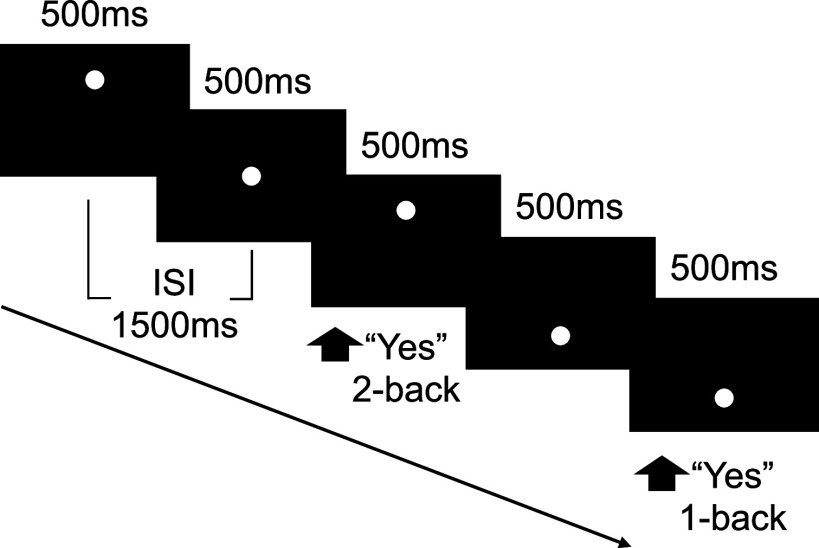
Task design of the spatial N-back working memory paradigm. Illustration of the N-back task structure, highlighting the sequence and timing of stimulus presentation and response requirements for 1-back and 2-back conditions. The inter-stimulus interval (ISI) is indicated.

The N-back WM task comprised 8 blocks: 3 for 0-back, 3 for 1-back, and 2 for 2-back loads (as depicted in Figure 1). Stimuli were white dots displayed at varying positions along the vertical midline of the monitor with equal probability. Each stimulus was visible for 500 ms, followed by a 1500 ms inter-stimulus interval. For the 0-back condition, participants were asked to press a key when the stimulus appeared. For the 1-back and 2-back conditions, participants were instructed to respond based on the position of the dot relative to the one or two preceding trials, respectively. The order of block presentation was randomized to prevent consecutive repetition of the same load more than twice. The task lasted for 5 min and 48 s and began with a 10-s fixation period followed by a 16-s instruction period. Each block lasted 24 s, consisting of 12 trials. The experiment was administered using E-Prime 1.1 software (https://www.pstnet.com/products/e-prime/).

### Behavioral data analyses

Accuracy and reaction time (RT) for the N-back task were recorded for each load, and repeated-measures Analysis of Covariance (ANCOVA) was applied to these variables. The signal-detection sensitivity *d*′ measure was also employed to evaluate the performance of WM. *d*′ was calculated as *Z*_hit_ − *Z*_false alarm_, with ‘hit’ denoting the correct response rate to target trials and ‘false alarm’ (FA) representing incorrect responses to nontarget trials (Haatveit et al., 2010). Higher *d*′ value signifies better sensitivity of the participant to discriminate target from nontarget and therefore means better WM capability. ANCOVA was applied to test the between-group difference in *d’* with age as a covariate. Considering the matching of gender was sufficiently robust to minimize potential confounding effects, we used age as a covariate in the analysis to account for potential subtle and continuous effects of age-related variance on cognitive performance.

### Imaging acquisition and preprocessing

Imaging data were acquired using a Philips Achieva X-series 3.0 Tesla Magnetic Resonance Imaging (MRI) scanner at the Affiliated Brain Hospital of Guangzhou Medical University. Structural imaging parameters included TE = 3.8 ms, TR = 8.3 ms, FOV = 256 mm × 256 mm, acquisition matrix = 256 × 256, and a reconstruction voxel size of 1 × 1 × 1 mm^3^. Functional MRI (fMRI) was performed using a T2*-weighted single-shot echo-planar imaging sequence with TR = 2 s, TE = 30 ms, FOV = 224 mm × 224 mm, matrix = 64 × 64, 33 interleaved slices, and 174 scans collected.

fMRI data were preprocessed using SPM12 (https://www.fil.ion.ucl.ac.uk/spm/) and DPABI toolbox (Yan, Wang, Zuo, & Zang, 2016) on Matlab (R2019b). The following preprocessing steps were performed: slice timing correction, motion correction, co-registration of the functional images to the structural image, spatial normalization to MNI space, and smoothing with a Gaussian kernel (FWHM = 6 mm).

### Brain activation analyses

In the first-level analysis of the N-back task, the general linear model (GLM) was used to obtain the activations of N-back under each condition for each participant. The default six head motion parameters were included as regressors of no interest. Group-level analyses were conducted using repeated-measures ANCOVA on the N-back activation maps to investigate group effects. Statistical analyses were age-adjusted, and statistical threshold was set at height threshold of *p* < 0.005 and extent threshold of *p* < 0.05, with cluster-level Gaussian Random Field (GRF) correction and gray matter mask applied. Post-hoc analyses were further performed on the estimated contrasts extracted from significant brain areas. For the brain areas showing significant group effects, the correlation between their activations and behavior performance was examined by Pearson correlation analyses with age as covariate.

### Psychophysiological interaction

Voxel-wise psychophysiological interaction (PPI) analysis was conducted with the above resultant cluster as region of interest (ROI) in order to assess the condition-dependent synchronization between the ROI and other brain areas. Additionally, bilateral dorsolateral prefrontal cortex (DLPFC) ROIs (left DLPFC, MNI coordinates [*x, y, z*]: −44, 18, 22; and right DLPFC, MNI coordinates [*x, y, z*]: 40, 32, 30) were defined as these two areas showed strong responses to the N-back paradigms consistently in previous studies (Kowalczyk et al., 2021; Owen, McMillan, Laird, & Bullmore, 2005). The ROIs were defined as 10 mm radius spheres in the standard space. Subsequently, an independent samples t-test was applied to evaluate condition-dependent synchronization differences. Statistical analyses were age-adjusted, and statistical threshold was set at height threshold of *p* < 0.005 and extent threshold of *p* < 0.05, with cluster-level GRF correction and gray matter mask applied. For the condition-dependent connections showing significant between-group differences, their correlation with behavioral performance was examined by Pearson correlation analyses with age as covariate.

## Results

### Significantly poorer performance on the 1-back task in BD offspring

In the current study, 38% (18/47) of the BD offspring exhibited poor performance on the 2-back WM task, with accuracy falling below chance levels (≤50%). Approximately 13% (6/47) of the BD offspring performed poorly on the 1-back task (accuracy ≤50%). To ensure data quality, the study’s focus was narrowed to the 0-back and 1-back conditions. The final analysis comprised 41 BD offspring and 25 HCs, matched for age and gender (see details in Supplementary Table S1).

**Figure 2.**
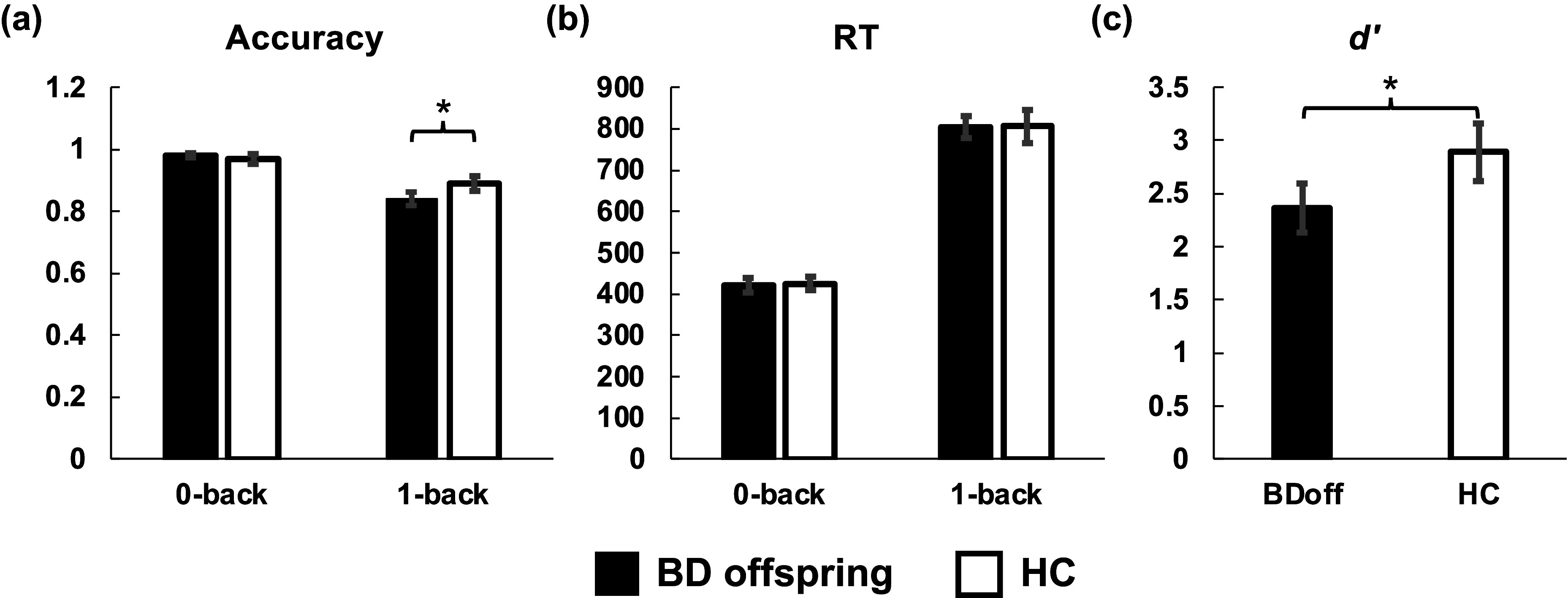
Behavioral performance in the N-back task. (a) Accuracy (ACC) across 0-back and 1-back conditions, demonstrating a significant interaction between task load and group. (b) Reaction time (RT) for the same conditions, with no significant group differences. (c) The *d*′ measure for 1-back task performance, showing significant between-group differences. *Indicates *p* < 0.05, Holm’s Bonferroni corrected for multiple comparisons, with age as a covariate.

A repeated-measures ANCOVA, with age as covariate, revealed no significant main effect of group on accuracy (*F*(1,63) = 2.62, *p* = 0.11), but a significant interaction between load and group (Figure 2a; *F*(1,63) = 4.45, *p* = 0.039) was observed. Post-hoc analysis, adjusted for multiple comparisons using Holm’s Bonferroni method, indicated a significant difference in 1-back task accuracy between the two groups (*p* = 0.029). RT did not differ significantly between groups (Figure 2b; *ps* > 0.05). An ANCOVA with age as a covariate showed a significant between-group difference in the *d’* measure for the 1-back task (Figure 2c; *F*(1,63) = 4.89, *p* = 0.031).

### Significant hyperactivation in the right occipital area during the N-back task in the BD offspring

**Figure 3.**
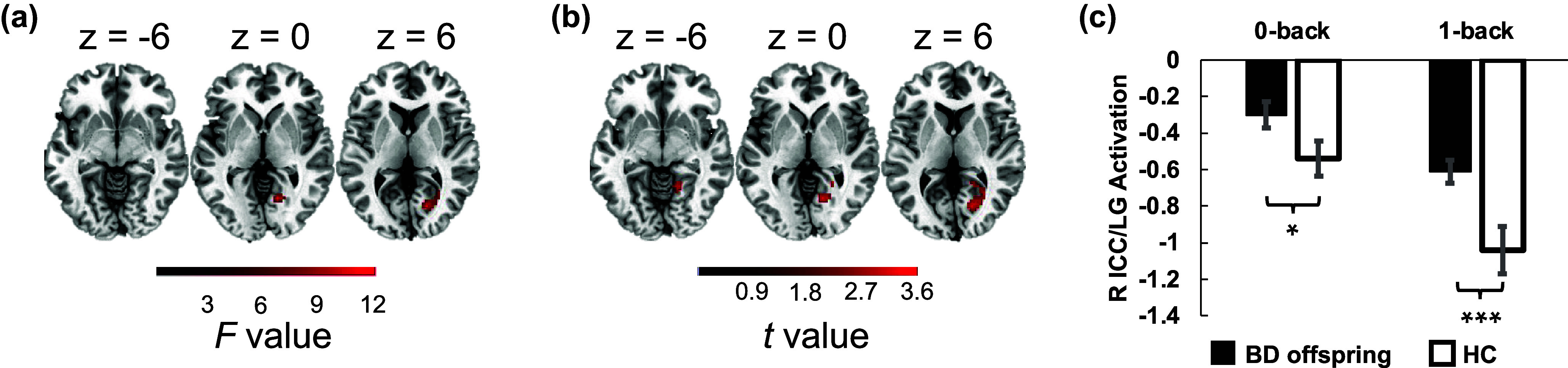
Group differences in brain activation during working memory task. (a) Activation in the right intracalcarine cortex/lingual gyrus (ICC/LG), showing a significant main effect of group. (b) and (c) Enhanced activation in the right ICC/LG during 0-back and 1-back tasks in BD offspring compared to HCs. Abbreviations: ICC, intracalcarine cortex; LG, lingual gyrus; R, right. *, **, *** indicate *p* < 0.05, *p* < 0.01, *p* < 0.001, respectively, Holm’s Bonferroni corrected for multiple comparisons in post-hoc analyses, with age as a covariate.

Repeated-measures ANCOVA with age as a covariate was conducted to compare the between-group differences in the brain activation during the N-back task. The results disclosed a significant main effect of group in the right intracalcarine cortex/lingual gyrus (ICC/LG; Figure 3a and Supplementary Table S2). Positive effects of group showed significant hyperactivation in the right ICC/LG during both 0-back and 1-back load conditions in BD offspring compared to HCs (Figure 3b and Supplementary Table S3), which was evident in the further post-hoc analyses on the extracted estimated contrast (Figure 3c).

We also conducted a repeated-measures ANCOVA including the 2-back task (see Supplementary Results). The results revealed significant main effects of group in the bilateral lingual gyrus (LG), right supramarginal gyrus (SMG), and right superior temporal gyrus (STG) (Supplementary Figure S1a), and a significant group x load interaction was identified in the left frontal pole (FP) (Supplementary Figure S1b). ROI-based analyses revealed hyperactivation in the bilateral LG and right STG and hypoactivation in the right SMG in BD offspring compared to HCs during the task (Supplementary Figure S1c–f). Additionally, the left FP showed hyperactivation in BD offspring under the 2-back condition but reduced activation compared to HCs under the 0-back condition (Supplementary Figure S1g).

### Suppressed condition-dependent connectivity in the BD offspring when memory load increased

**Figure 4.**
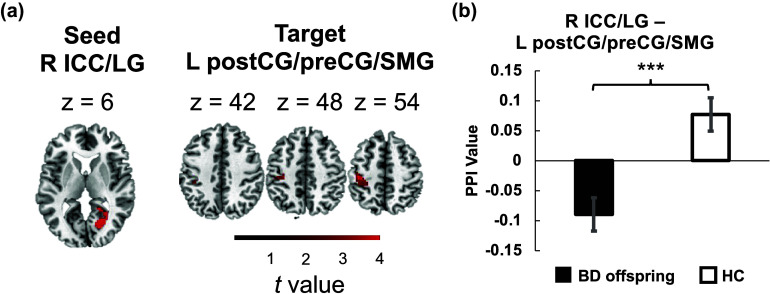
Psychophysiological Interaction (PPI) analysis revealing connectivity differences. (a) and (b) PPI analysis demonstrating significantly higher condition-dependent connectivity between the right ICC/LG and the left postcentral gyrus/precentral gyrus/supramarginal gyrus (postCG/preCG/SMG) in the BD offspring group compared to the HC group during the transition from 0-back to 1-back tasks. Abbreviations: ICC, intracalcarine cortex; LG, lingual gyrus; postCG, postcentral gyrus; preCG, precentral gyrus; SMG, supramarginal gyrus; L, left; R, right.

Next, we conducted voxel-wise PPI analysis following the identification of the right ICC/LG cluster. The results revealed significant suppression of connectivity between the right ICC/LG and the left postcentral gyrus/precentral gyrus/supramarginal gyrus (postCG/preCG/SMG) in BD offspring compared to HCs during the transition from 0-back to 1-back tasks (Figure 4a and Supplementary Table S4). This suppression was evident in the PPI maps from independent-sample *t*-tests (Figure 4b).

We also conducted an additional PPI analysis with a 10-mm spherical ROI centered on the right ICC/LG. The analysis identified the same postcentral areas, although the result did not survive GRF correction (see Supplementary Results).

**Figure 5.**
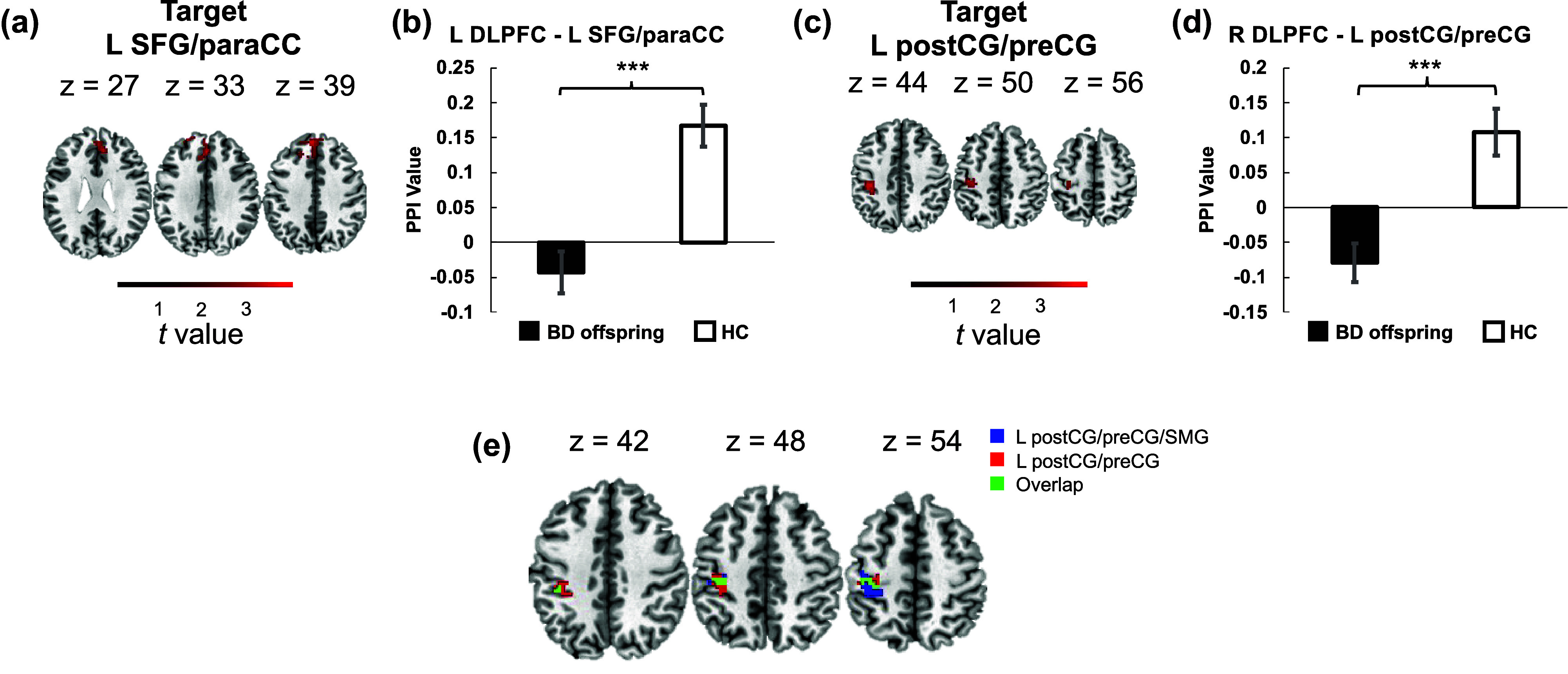
PPI analyses of DLPFC connectivity during task load transition. (a) Targeted area of the left dorsolateral prefrontal cortex (DLPFC). (b) Significant difference in condition-dependent connectivity between the left DLPFC and the left superior frontal gyrus/paracingulate cortex (SFG/paraCC) in BD offspring compared to HCs. (c) Targeted area of the right DLPFC. (d) Significant difference in condition-dependent connectivity between the right DLPFC and the left postcentral gyrus/precentral gyrus (postCG/preCG) in BD offspring compared to HCs. (e) Overlapping central areas (green) between the regions targeted by the right ICC/LG (blue) and the right DLPFC (red). Abbreviations: DLPFC, dorsolateral prefrontal cortex; paraCC, paracingulate cortex; SFG, superior frontal gyrus.

We additionally examined condition-dependent connectivity of bilateral DLPFC ROIs (left DLPFC, MNI coordinates [*x, y, z*]: −44, 18, 22; and right DLPFC, MNI coordinates [*x, y, z*]: 40, 32, 30). These two areas showed strong responses to the N-back paradigms consistently in previous studies (Kowalczyk et al., 2021; Owen et al., 2005). PPI analyses indicated significantly suppressed condition-dependent connectivity between the left DLPFC and the left superior frontal gyrus/paracingulate cortex (SFG/paraCC) and that between the right DLPFC and the left postcentral gyrus/precentral gyrus (postCG/preCG) in BD offspring compared to HCs when the memory load increased from 0-back to 1-back (Figure 5a–d and Supplementary Table S4). Interestingly, there was an overlap in the target postcentral region of the right DLPFC and the target postcentral region of the right ICC/LG (Figure 5e).

### Decorrelation between condition-dependent connectivity of the right occipital area and WM in BD offspring

**Figure 6.**
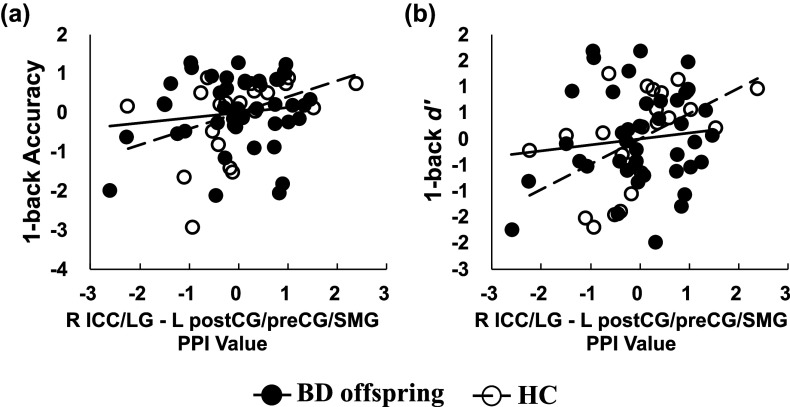
Correlation between brain connectivity and behavioral performance. Condition-dependent connectivity between the right ICC/LG and left postCG/preCG/SMG was positively correlated with (a) accuracy and (b) *d*′ of the 1-back task in HCs but not in BD offspring. Age was used as a covariate. Abbreviations: ACC, accuracy; ICC, intracalcarine cortex; LG, lingual gyrus; postCG, postcentral; preCG, precentral gyrus; SMG, supramarginal gyrus.

Correlation analyses were conducted to explore the relationship between the N-back task performance and the activation and condition-dependent connectivity that exhibited between-group differences. In the HC group, condition-dependent connectivity between the right ICC/LG and left postCG/preCG/SMG was positively correlated with both accuracy (Figure 6a; for HC: r = 0.41, *p* = 0.049) and *d’* (Figure 6b; for HC: *r* = 0.52, *p* = 0.010) in the 1-back task (Figure 6). However, these correlations were absent in the BD offspring (*ps* > 0.05). No significant correlations were identified for other brain characteristics that displayed between-group differences (*ps* > 0.05).

## Discussion

The present study delved into the WM deficits and the neural substrates underpinning these deficits in offspring of individuals with BD offspring. Our findings are in concert with the existing literature, revealing that BD offspring participants exhibited diminished performance on the 1-back task, characterized by lower accuracy and *d*′ in comparison to HCs. Notably, we observed hyperactivation in the right ICC/LG during the N-back task in BD offspring. Furthermore, we identified a suppression in condition-dependent connectivity between the right ICC/LG and the left postCG/preCG/SMG as the task load increased from 0-back to 1-back in BD offspring. Additionally, we noted suppressed condition-dependent connectivity between the left DLPFC and the left SFG/paraCC, as well as between the right DLPFC and the left postCG/preCG, under higher task loads in BD offspring.

WM, a fundamental cognitive function, is essential for a myriad of daily activities. Patients with BD have consistently demonstrated WM deficits, regardless of their emotional state (Soraggi-Frez et al., 2017). Given the pronounced heritability of BD, it is noteworthy that first-degree relatives of BD patients, who do not exhibit the disorder, also present with WM deficits, as corroborated by our study (Soraggi-Frez et al., 2017; Valencia-Echeverry et al., 2022). This convergence of evidence posits WM deficits as a potential early marker of BD.

Our results indicated abnormal hyperactivation in the right ICC/LG among BD offspring during the N-back task, a finding that resonates with studies implicating the impairments in the sensory system in BD. For example, resting-state functional connectivity (rsFC) studies have linked higher polygenic risk for BD with increased rsFC of the visual network with the left ICC (Jiang et al., 2023). The hyperactivation observed may reflect a compromised efficiency in the primary visual area during visual stimulus processing, even in asymptomatic BD offspring. The suppression of condition-dependent connectivity between the right ICC/LG and the left sensorimotor area (postCG/preCG/SMG) during task load increased in BD offspring, as opposed to HCs, suggesting an impairment in sensory information processing and integration. Furthermore, the deficits in such psychomotor systems have been proposed to result in the impairment in connecting with the external environment and setting the exteroceptive input/somatomotor output processing in the BD patients. Thus, the current results might indicate the deficits in psychomotor systems as an early indicator for the disease and potentially undermine WM function in BD offspring (Magioncalda & Martino, 2022; Martino & Magioncalda, 2022).

Although no significant abnormal frontal activation was detected, the condition-dependent connectivity within the prefrontal system and between the primary sensory and higher cognitive systems was compromised in BD offspring. The DLPFC, a region integral to higher cognitive functions including WM, has been associated with WM deficits in BD (Townsend et al., 2010). The cingulofrontal system has been implicated to be tightly associated with affective disorder, especially BD psychology (Acuff et al., 2018; Lin et al., 2020; Sun et al., 2019). Prior studies have identified cortical thickness reduction in the paraCC area as a morphological signature of BD since such area played a critical role in executive functions as a cingulofrontal transition zone (Bush et al., 2002; Fornito et al., 2008; Vogt, Nimchinsky, Vogt, & Hof, 1995). Our findings suggest a disruption in the information transfer and integration within the prefrontal system in BD offspring, even when activation levels are comparable to HCs. The reduced connectivity between the right DLPFC and left post/preCG may signal an interruption in the integration of information between higher cognitive and sensorimotor systems. Intriguingly, the overlap between the target regions of the right DLPFC and the right ICC/LG that showed hyperactivation points to a potential impairment in the pathway connecting primary visual, sensorimotor, and higher cognitive systems.

Previous literature has suggested the critical role of the impairments in executive and motor functions in BD. Cognitive dysfunction, particularly in psychomotor and executive functioning, has been considered an intermediate cognitive phenotype in a subset of individuals who later developed BD (Langenecker et al., 2010). Moreover, impairments in executive and motor functioning were linked to deficits in WM during the early stages of the illness in first-episode psychosis (Pérez-Iglesias et al., 2010). Additionally, it has been suggested that the visual system integrates sensory information that is relayed to higher-order executive processes, which is associated with emotion perception and WM (Chang et al., 2019; Le et al., 2017). All these findings may highlight the impairments in information integration between the sensory system and the higher-order association system in BD offspring with subthreshold symptoms, suggesting the potential target for early diagnosis and intervention (Lin et al., 2015, 2018).

Several limitations warrant consideration in our study. Firstly, the unequal group sizes, despite matched age and gender, may introduce potential biases. Secondly, the poor performance of BD offspring on the 2-back task precludes a deeper exploration of neural abnormalities associated with varying WM loads. Thirdly, although different ROI types of right ICC/LG exhibited similar results, the inconsistent types of ROIs used in the PPI analyses present another limitation. Lastly, the direction of the abnormal information flow found in the current results remains uncharted, necessitating further investigation with non-invasive brain stimulation techniques to elucidate the underlying mechanisms.

Our findings underscore the presence of WM deficits, hyperactivation of the right ICC/LG, and suppressed condition-based connectivity in BD offspring during an N-back task. These impairments suggest inefficiencies in information processing and transfer within the primary sensory system. Additionally, we observed deficits in the interaction within the prefrontal system and between the primary sensory and higher-order cognitive systems. These insights offer potential targets for early detection and intervention strategies for individuals at risk for BD.

## Supporting information

Xie et al. supplementary material Xie et al. supplementary material

## Data Availability

The data that support the findings of this study are available on request from the corresponding author.

## Supplementary material

The supplementary material for this article can be found at http://doi.org/10.1017/S0033291725001060.
