# Supplementary material for: Cognitive and neural abnormalities: working memory deficits in bipolar disorder offspring
Source: Psychol Med. 2025 May 2;55:e130. doi: 10.1017/S0033291725001060 (PMC12094646; doi:10.1017/S0033291725001060)
Supplement: Xie et al. supplementary material [file S0033291725001060sup001.docx]

**Supplementary Results**

**Repeated ANCOVA analyses of sub-dataset with good 2-back performance**

An analysis including the 2-back task was conducted. After excluding participants with 2-back task accuracy at or below chance level (≤ 50%), the final sample consisted of 28 for BD offspring and 19 healthy controls (HCs). Repeated measures ANCOVA were performed on accuracy, reaction time and also brain activations, while ANCOVA was conducted on *d’*. Although BD offspring showed lower behavioral performance, these differences did not reach statistical significance (*ps* > 0.182, Bonferroni-corrected).

For neural activation patterns, statistical analyses were age-adjusted and statistical threshold was set at height threshold of p < 0.005, and extent threshold of p < 0.05 with cluster-level GRF correction and gray matter mask applied. Significant main effects of group were observed in the bilateral lingual gyrus (LG), right supramarginal gyrus (SMG) and right superior temporal gyrus (STG) (Supplementary Fig. 1A). Additionally, a significant group x load interaction was identified in the left frontal pole (FP) (Supplementary Fig. 1B). ROI-based analyses revealed hyperactivation in the bilateral LG and right STG, alongside hypoactivation in the right SMG in BD offspring compared to HCs during the task (Supplementary Fig. 1C-1F). furthermore, in the left FP, BD offspring exhibited hyperactivation under the 2-back condition but reduced activation relative to HCs under the 0-back condition (Supplementary Fig. 1G).

Despite the limited sample size, these findings provide further evidence of neural abnormalities in BD offspring, particularly in brain regions associated with psychomotor and higher-order cognitive processing.


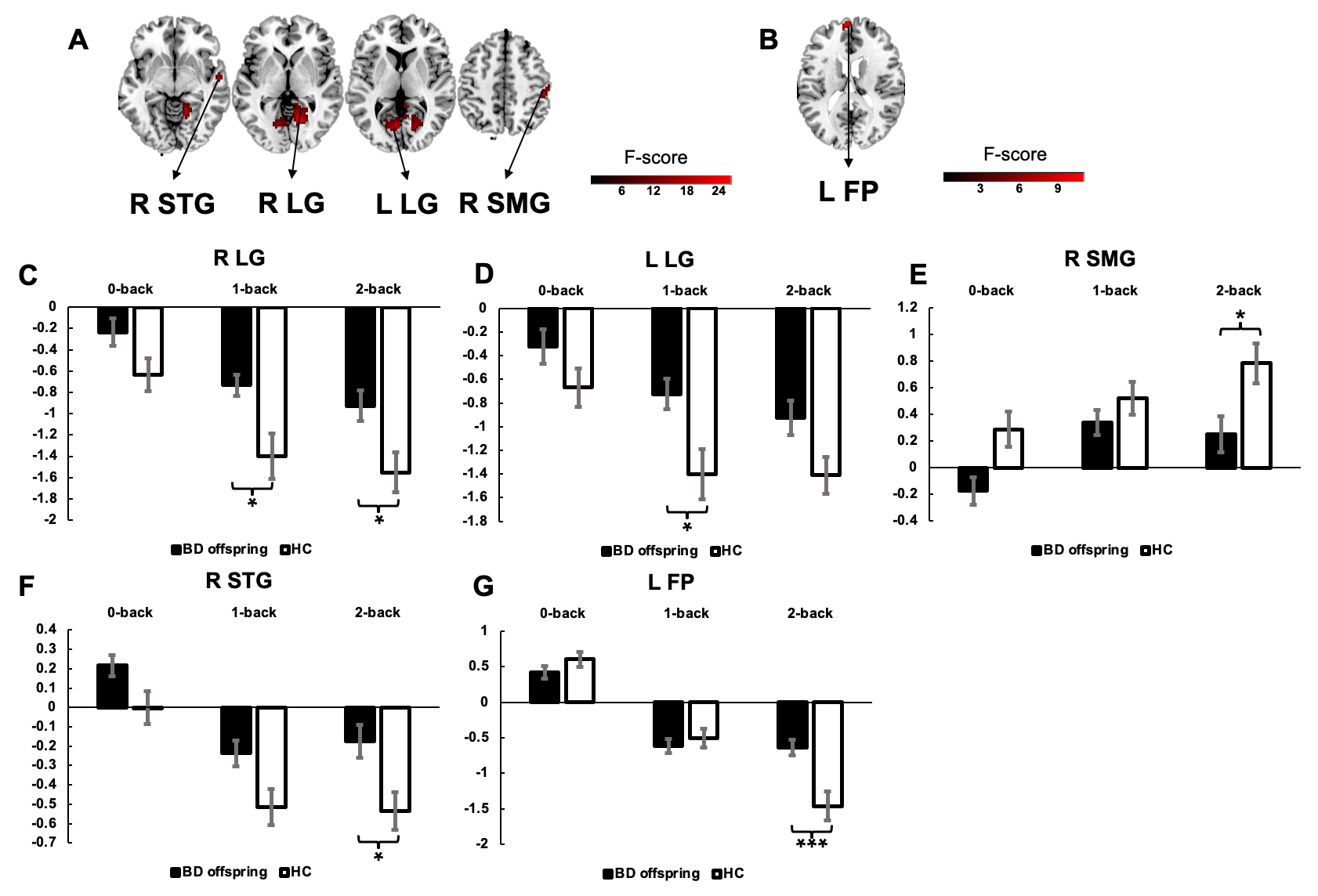


Supplementary Figure 1. Results of repeated measures ANCOVA (group: BD offspring, HC; load: 0-back, 1-back, 2-back) with age as a covariate. (A) Brain areas showing a significant main effect of group. (B) Brain areas showing a significant group x load interaction. (C)-(G) ROI-based analyses of brain regions with significant effects, illustrating group differences in activation patterns across task conditions.

**PPI analysis result of 10-mm spherical ROI centered in the right ICC/LG**

A PPI analysis was conducted with a 10-mm spherical ROI centered in the right ICC/LG (MNI coordinate: 18, -72, 6, identified from the ANOVA analysis on brain activation). The results revealed reduced condition-dependent connectivity with the left postCG/preCG/SMG (MNI coordinate: -39, -24, 54; peak *t* value = 3.94, 51 voxels, height threshold *p* < 0.005) in BD offspring as memory load increased. This cluster was consistent with the findings from the PPI analysis using right ICC/LG cluster as the ROI but did not survive GRF correction.

Supplementary Table 1. demographic characteristics.

|  | BD offspring  (n = 41) | HC  (n = 25) | Between-group differences |
| --- | --- | --- | --- |
| Age (years) | 16.98 (4.37) | 15.43 (1.29) | t(64) = 561.50, *p* = 0.521^a^ |
| Gender (male: female) | 15:26 | 9:16 | $\chi^{2}$ = 0.002, *p* = 0.962^b^ |

Values are mean (standard deviation). Abbreviation: BD offspring, offspring of parents with bipolar disorder; HC, healthy controls.

^a^ Mann-Whitney U test, ^b^ chi-square test.

Supplementary Table 2. Significant brain regions in 2-way ANOVA analysis.

| Region | Cluster Size  (voxels) | *F* | Peak MNI Coordinate  mm (x, y, z) |
| --- | --- | --- | --- |
| ***Main effect of group*** |  |  |  |
| R ICC/LG | 52 | 13.95 | 18, -72, 6 |
| ***Group x Load interaction*** |  |  |  |
| No brain region |  |  |  |

Voxel-wise threshold *p* < 0.005, cluster-level GRF corrected *p* < 0.05. Age adjusted and gray matter mask applied. Abbreviation: ICC, intracalcarine cortex; LG, lingual gyrus; R, right.

Supplementary Table 3. Brain regions showed positive effect of group in brain activation

| Region | Cluster Size  (voxels) | Peak t-value | Peak MNI Coordinate  mm (x, y, z) |
| --- | --- | --- | --- |
| ***Positive effect of BD offspring*** |  |  |  |
| R ICC/LG | 129 | 3.73 | 18, -72, 6 |
| ***Positive effect of HC*** |  |  |  |
| No brain region |  |  |  |

Voxel-wise threshold *p* < 0.005, cluster-level GRF corrected *p* < 0.05. Age adjusted and gray matter mask applied. Abbreviation: ICC, intracalcarine cortex; LG, lingual gyrus; R, right.

Supplementary Table 4. Brain regions with significant between-group difference in PPI analyses.

| Region | Cluster Size  (voxels) | Peak t-value | Peak MNI Coordinate  mm (x, y, z) |
| --- | --- | --- | --- |
| ***Target: R ICC/LG*** |  |  |  |
| L postCG/preCG/SMG | 80 | 4.24 | -39, -24, 54 |
| ***Target: L DLPFC*** |  |  |  |
| L SFG/paraCC | 149 | 3.96 | -3, 48, 39 |
| ***Target: R DLPFC*** |  |  |  |
| L postCG/preCG | 84 | 3.97 | -39, -27, 48 |

Voxel-wise threshold *p* < 0.005, cluster-level GRF corrected *p* < 0.05. Age adjusted and gray matter mask applied. Abbreviation: DLPFC, dorsolateral prefrontal cortex; ICC, intracalcarine cortex; LG, lingual gyrus; paraCC, paracingulate cortex; postCG, postcentral gyrus; preCG, precentral gyrus; SFG, superior frontal gyrus; SMG, supramarginal gyrus; L, left; R, right.
